# A New Approach to Develop Resistant Cultivars Against the Plant Pathogens: CRISPR Drives

**DOI:** 10.3389/fpls.2022.889497

**Published:** 2022-04-27

**Authors:** Mumin Ibrahim Tek, Kubra Budak

**Affiliations:** ^1^Molecular Mycology Laboratory, Plant Protection Department, Akdeniz University, Antalya, Turkey; ^2^Plant Transformation Laboratory, Plant Biotechnology, Akdeniz University, Antalya, Turkey

**Keywords:** CRISPR, breeding, gene drive, plant pathogen, plant resistance, CRISPR drive, plant disease, gene editing

## Abstract

CRISPR drive is a recent and robust tool that allows durable genetic manipulation of the pest population like human disease vectors such as malaria vector mosquitos. In recent years, it has been suggested that CRISPR drives can also be used to control plant diseases, pests, and weeds. However, using a CRISPR drive in *Arabidopsis* for the first time in 2021 has been shown to use this technology in plant breeding to obtain homozygous parental lines. This perspective has proposed using CRISPR drive to develop pathogen-resistant cultivars by disrupting the susceptibility gene (*S*). In the breeding program, CRISPR is used to create *S*-gene mutations in two parental lines of hybrid cultivars. However, CRISPR must be reapplied or long-term backcrossed for the parental line to obtain homozygous *S*-mutant cultivars. When a parental line crosses with different parental lines to develop new hybrids, heterozygous *S*-mutations could not resist in hybrid against the pathogen. CRISPR drives are theoretically valid to develop homozygous *S*-mutant plants against pathogens by only routine pollination after CRISPR drive transformation to just one parental line. This way, breeders could use this parental line in different crossing combinations without reapplying the genome-editing technique or backcrossing. Moreover, CRISPR drive also could allow the development of marker-free resistant cultivars with modifications on the drive cassette.

## Introduction

Gene drives are suggested at the beginning of the last century to change the population’s genetic structure. Initial gene drives were based on homing endonuclease genes (HEGs), which cleave 20–30 nucleotide-sized recognition sites on chromosomes. After cleavage on target, DNA is repaired with homologous directed repair (HDR). During repair, the HEG allele is used as the template, and HEG-carrying homozygotes are formed on both alleles of the organism (Burt, 2003). In this way, HEGs are inherited in every generation within the population, and therefore it is called Super-Mendelian segregation (Chevalier and Stoddard, 2001; Goddard et al., 2001). HEG-based drives are created by integrating HEG and an interesting gene with a promoter. When this drive is included in 1% of the population, 90% of the population contains gene drive after 12 generations (Burt, 2003).

Gene drives allow the knockout of an existing gene or the inclusion of a different gene in the population. In each generation, this knockout or transgene spreads through the population. Moreover, the gene drive could be programmed to create lethal mutations, sex ratio disruptions, and different manipulations of the pest populations (Wood and Newton, 1991; Huang et al., 2007; Deredec et al., 2008).

Homing endonuclease gene-based gene drives have generally been designed only in laboratory mosquitoes and fruit flies (Chan et al., 2011; Windbichler et al., 2011; Simoni et al., 2014). Earlier, the homing endonuclease cleavage site was transferred to these organisms, and gene drive studies were carried out on these transgenic organisms. Although HEG drive is a powerful tool that can manipulate the population, the most significant lack of HEG-based gene drives is that the endonuclease recognition sites are not common naturally. Even though different study groups have designed different gene drives for pests, the application of these gene drives was limited due to the target region problem, but CRISPR/Cas9 brought a new solution to this problem (Burt and Koufopanou, 2004; Schliekelman et al., 2005; Sinkins and Gould, 2006; Bax and Thresher, 2009; Medina, 2018).

There have been significant developments in gene drives with the CRISPR era. CRISPR drives could be used to knock out a target gene or introduce a different gene, such as HEG drive. Moreover, the CRISPR drive could be directed to the target sequence in an organism. The Cas9 endonuclease cuts the target site in the organism’s genome with guide RNA recognition, and the CRISPR drive is used as a template during HDR. The drive-cassette is copied into the first allele with HDR. Cas9 in the drive-cassette expresses and cuts the second allele. The drive-cassette on the first allele is used as a template during HDR for the second allele. In each generation, the drive-cassette activates and spreads through mating in populations. In this way, target genes are knocked out, spreading in the population; different genes (payload) could be integrated with the CRISPR drive, the gene is attached to the organism genome in the target region, and the gene frequency is increased in the population (Esvelt et al., 2014). In recent years, CRISPR drives have been used on fruit flies (Gantz and Bier, 2015), mosquitoes (Gantz et al., 2015; Hammond et al., 2016), mice (Grunwald et al., 2019), yeast (Dicarlo et al., 2013; Yan and Finnigan, 2018), and *Arabidopsis* (Zhang et al., 2021).

CRISPR drivers have often been suggested to be used to control pest populations through genetic manipulation. However, the first application of CRISPR drives in *Arabidopsis* may have expanded the use of CRISPR drives (Zhang et al., 2021). Especially in plant breeding, CRISPR drives can be used to obtain homozygous parental lines and fix a specific characteristic on these parental lines (Siddiqui et al., 2021). In addition to plant breeding, this technology could contribute to the CRISPR gene-editing technique used in breeding programs to develop resistant cultivars. A CRISPR drive could be programmed to knock out a disease susceptibility (S) gene. The resistant parental lines are obtained due to a mutation on the S gene, and the parental line can be used in different hybrid combinations. Thus, resistant mutant cultivars can be developed in a shorter time than the CRISPR gene-editing technique. This perspective has focused on using CRISPR drives to develop resistant cultivars in a shorter time and more efficiently in plant breeding.

## Subsections

### Gene Drives in Agriculture

Gene drive studies have focused on eradicating vectors that carry diseases that threaten human health, such as *Anopheles gambiae*. However, gene drive has also been shown in agriculture with the CRISPR era. Alternative solutions were proposed to use CRISPR drive in the management of plant pathogens, pests, and weeds (Medina, 2018; Neve, 2018; Barrett et al., 2019; Gardiner et al., 2020).

Using local CRISPR drives has been suggested for the control of pests rather than global gene drives designed to destroy a population by distorting the sex ratio of the pest population or by transferring a lethal gene. Local gene drives, also known as sensitizing drives, can be used to sensitize a population to pesticides. When sensitizing drives carrying pests are released, the drive quickly spreads throughout the population. The gene drive spreads to most of the part population, the pesticide is applied, and the sensitive population is eradicated in a local area. Like this strategy, the drive may also be programmable to reverse pesticide resistance in pests. Moreover, the plant pathogen–vector relationship can be manipulated, and vector-borne disease can be prevented in the field (Esvelt et al., 2014; Medina, 2018).

Fungal plant pathogens are also controlled by using gene drives. For instance, a SpokI-based gene drive was designed to knock out two virulence loci of the critical wheat pathogen, *Fusarium graminearum*, and the pathogen’s virulence is reduced *in vitro* conditions with this drive (Gardiner et al., 2020). Gene drives could be used to manipulate weed competitive capacity. For example, *Rht1*, which causes dwarf in wheat, could be included in weeds populations from the Poaceae family and may reduce the competitive capacity of the weeds. Additionally, a CRISPR drive can be designed to manipulate the sex ratio in dioecious weeds such as *Amaranthus*. Therefore, pollen or ovule formation is deflected in the weed population. Another strategy for weed control is sensitization drives for the control of pests. With the sensitization drive, herbicide resistance could be reversed in weed species that have developed resistance to the herbicide (Neve, 2018; Barrett et al., 2019).

### CRISPR Drives Used in Plant Breeding

Plant susceptible (*S*) genes are coding proteins that could cause recognition of the host by the pathogen, negative regulation of the plant defense system, and pathogen penetration facilitation (van Schie and Takken, 2014). By using CRISPR, mutations could be induced on the genes to knock out the *S*-genes by non-homologous end joining (NHEJ) and develop new resistant cultivars against plant pathogens. For commercial usage, CRISPR is applied to each parental line, and two *S*-mutant parental lines are obtained. The homozygous *S*-mutant parental lines are crossed, resistant cultivar seeds have high yielding, and high-quality hybrids are developed. CRISPR also allows the development of transgene-free mutant plants (He and Zhao, 2020).

Although the *S*-mutant parental line is obtained by CRISPR, this parental line cannot be used directly in different hybrid combinations to develop resistant cultivars against the pathogen. Because homozygous *S*-mutations confer complete resistance and when the *S*-mutant parental line is crossed with another non-mutant parental line (having high quality and yielding, or other superior features), the heterozygous mutation could not show resistance in the hybrid. Thus, CRISPR must be reapplied for each different hybrid parental line, or a long-term backcross must be carried out to acquire resistance to cultivars against the pathogen. While traditional hybrid breeding takes 8–10 years, CRISPR has reduced this time to 4–6 years to develop resistant cultivars (Chen et al., 2019). Although CRISPR could use existing hybrid cultivars to acquire resistance against pathogens with *S*-gene knockout, developing different cultivars in the other breeding programs with CRISPR is still challenging and time-consuming. CRISPR drives could be an alternative solution to solve these problems.

CRISPR drive has been used first in the model plant *Arabidopsis thaliana*. A CRISPR drive targeting *CRYPTOCHROME 1* (*CRY*1) was designed in the study. The results showed that the CRISPR drive could convert the heterozygous allele into a homozygous one. Although the *Arabidopsis* plants’ HDR rate was low (3–8%), when these plants were crossed with wild-type (WT) *Arabidopsis*, the resulting plants showed the mutant-*CRY*1 phenotype (Zhang et al., 2021). The study shows that CRISPR drive can be used in plant breeding to obtain a homozygous parental line or fix a feature on the parental line. In plant-breeding programs, 7–8 generations are inbred to obtain parental homozygous lines with different features. These parental lines are crossed in various combinations to obtain hybrid cultivars with different characteristics. The study by Zhang et al. (2021) has shown that CRISPR drive could significantly reduce the inbreeding process to obtain homozygous parental lines (Siddiqui et al., 2021).

The efficient HDR gene drive delivery method was unknown in plants until the first use of CRISPR drives in *Arabidopsis*. The study has shown that the CRISPR drive could convert the heterozygous allele into a homozygous. CRISPR drive can be used in plant breeding to obtain homozygous lines or fix a feature on the parental line. It has been suggested that CRISPR drive can significantly reduce the inbreeding process time (Siddiqui et al., 2021).

A resistant *S*-mutant parental line could be obtained with CRISPR drive, and this line could be included directly in different breeding programs without additional CRISPR application or backcross. In this way, repetitive challenging processes (transformation, regeneration, and acclimatization) could be eliminated, and homozygous *S*-mutation could be transferred to different hybrid cultivars with a routine and straightforward pollinating process.

### CRISPR Drive for Generating Resistant Cultivars Against Pathogens

**FIGURE 1:**
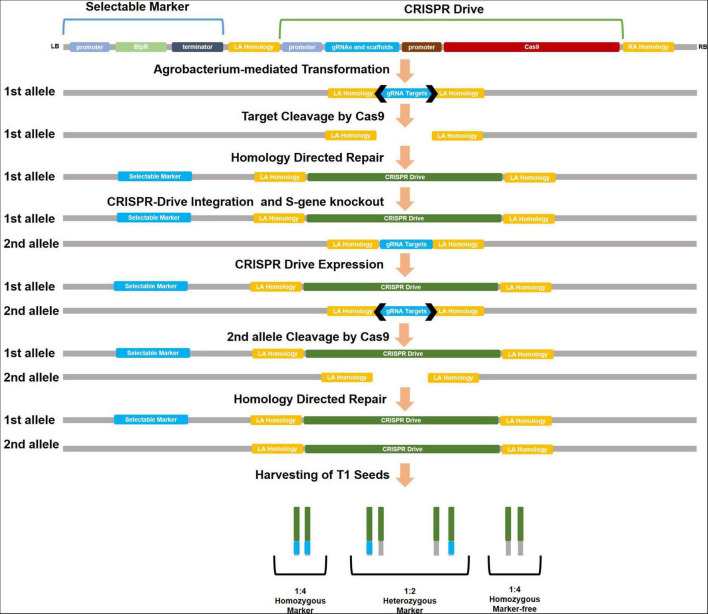
Development of *S*-mutant plants with CRISPR drive and schematization of drive cassette. Using disruption of meiotic control 1 (DMC1) and egg cell-specific (EC) promoters for Cas9 in drive cassette can increase HDR activity in the plant. Selectable marker (antibiotic/herbicide resistance, fluorescent, or different reporters) is cloned outside of homology arms (LA-RA homology) in CRISPR drive cassette. Thus after the cleavage at the second allele, selectable marker coding fragments could not be integrated into the second allele during HDR.

CRISPR drive contains a similar process of transferring new genes to plants by CRISPR. An S gene associated with disease susceptibility was selected as the target. The gRNAs that target the S gene in the plant are determined using different software (CRISPR-GE, CRISPOR, etc.). The specific gRNAs should not have an off-target effect, which is essential for the experiment’s effectiveness and ecological concerns. The cassette could consist of Cas9, gRNAs, and a meiosis-specific promoter for these components. Homology arms (left–right) compatible with the target region are added to both ends of the cassette. A selectable marker could be added to the cassette outside the homology arms. Transformation is performed on the plant after the cassette has been constructed into a plasmid (Figure 1).

**FIGURE 2:**
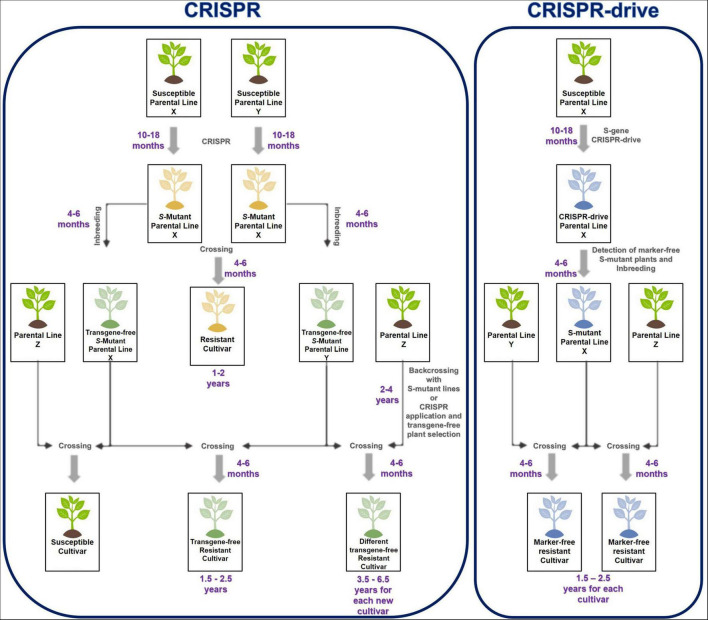
Comparison of CRISPR and CRISPR drive process to develop *S*-mutant cultivars against pathogens. CRISPR lets to generate resistant cultivars in a shorter time than traditional plant breeding. When CRISPR is used for new cultivars and transfers *S*-gene mutation to the cultivars, CRISPR must be reapplied to acquire resistance against pathogens. Alternatively, backcrossing is carried out to gain resistance from another parental line (Z). However, CRISPR drive could eliminate repetitive CRISPR application and backcross process when CRISPR drive carrying parental line (blue-X) pollinated with different parental lines (Y or Z), CRISPR drive actives and Cas9 cuts second *S*-allele on another parental line. CRISPR drive has copied itself on the second *S*-allele during HDR and disrupted the *S*-alleles. Only homozygous *S*-mutants plants are obtained from the result of crossing between these parental lines.

T1 seeds are harvested from the CRISPR drive carrying the parental line. From T1 plants, *S*-mutants and marker-free plants are selected. These marker-free mutants are verified for pathogen resistance. If the *S*-mutant parental line shows resistance against the pathogen, this line can be used directly as a source of resistance in plant-breeding programs. The *S*-mutant line was crossed with another line (WT) to obtain different hybrid plants; the homozygous *S*-mutation is observed in these plants. In this way, pathogen resistance could be rapidly acquired by different cultivars through pollination. Moreover, marker-free plants could be selected because only the first allele carries the antibiotic/herbicide resistance or fluorescent marker, while the CRISPR drive copied the second allele. Marker-free and drive-carrying *S*-mutant plants were selected in the segregated T1 generation at a 1:4 ratio (Figure 2).

## Discussion

CRISPR drives are powerful genome-editing tools that allow genetic modification in a population. Scientists have suggested using CRISPR drive as an alternative method to protect plants against diseases, pests, and weeds (Medina, 2018; Neve, 2018; Barrett et al., 2019). The CRISPR drive was first shown to work effectively in the model plant *Arabidopsis* last year (Zhang et al., 2021). The study showed that CRISPR drives are applicable in plant breeding. The results of the study are important for plant breeding. CRISPR drives could significantly shorten the process of obtaining long-term homozygous parental lines (Siddiqui et al., 2021). Additionally, the CRISPR drive theoretically solves the time and homozygosis problems, which is one of the biggest problems in integrating the CRISPR technique into commercial plant breeding.

Researchers in plant science often use CRISPR to characterize a gene or confer resistance to different stresses by inducing mutations in *S*-genes. Although CRISPR is a more economical, efficient, and short-term process than traditional breeding, there are problems in developing resistant hybrid cultivars with CRISPR. These issues are limiting the integration of CRISPR into plant breeding. When a homozygous *S*-gene mutation confers resistance against the pathogen, CRISPR should be performed on both parental lines of the commercial variety. These lines are obtained after 10–18 months of transformation, regeneration, and acclimatization. In the T1 generation, homozygous *S*-mutant plants are selected and inbred. Seeds are germinated and crossed to obtain *S*-mutant hybrid seeds. However, the breeder cannot use these parental lines to cross them with a different parental line for resistance against the pathogen. Because of the hybrid, the parental lines are crossed with different parental lines, do not contain homozygous *S*-mutations, and do not show resistance against a pathogen. For this, CRISPR should be reapplied to the parental lines of each commercial hybrid. Alternatively, the *S*-homozygous mutation was transferred to another parental line with a long-term backcross. Although CRISPR has some difficulties and disadvantages in its application, it provides the host with long-term, durable, and broad-spectrum resistance against a particular pathogen by knocking out the *S*-genes for resistance. At the same time, cultivating resistance in this way with CRISPR is 50–60% shorter than conventional breeding for resistance (Chen et al., 2019; Grunwald et al., 2019; Oliva et al., 2019).

The CRISPR drive is designed to knock out the *S*-gene that causes susceptibility to the plant pathogen. The *S*-mutant parental line obtained by CRISPR drive is crossed with other parental lines to develop high-yielding and quality-resistant cultivars against the pathogen. The parental line carrying CRISPR drive is used in different breeding programs as a source of resistance to obtaining new cultivars. The only routine crossing will yield *S*-gene mutant resistant cultivars (Figure 2). The CRISPR drive can also enable the development of marker-free cultivars. Even though CRISPR drive is GMO, at least marker-free plants could be developed to address these concerns about transgenic organisms and gene drives.

However, the most crucial problem with the CRISPR drive is low HDR efficiency in plants. In the repair of DSB, the NHEJ mechanism is more common than HDR in plants (Schiml and Puchta, 2016; Huang and Puchta, 2019). However, different methods have been developed to increase HDR activity in plants; using promoters such as disruption of meiotic control 1 (DMC1) and egg cell-specific (EC), HDR efficiency is increased (Klimyuk and Jones, 1997; Steffen et al., 2007; Sprunck et al., 2012; Miki et al., 2018; Wolter et al., 2018). Furthermore, the first CRISPR drive cassette has been constructed for *Arabidopsis* using two different promoters, and the DMC1 promoter was reported to produce higher HDR with 8% efficiency (Zhang et al., 2021).

Obtaining *S*-mutant plants with the drive significantly reduces the cost of CRISPR in plant breeding. Although CRISPR application is economical in developed countries, the costs are still relatively high in other countries. The CRISPR drive offers an additional strategy to reduce these costs. CRISPR drives have been developed in yeast, mosquitoes, fruit fly, and human cells, and the cost for preparing the drive construct is estimated to be around 1,000 dollars (Courtier-Orgogozo et al., 2017). Although the cost of preparing a construct for CRISPR is not very high, the repeated processes (transformation, regeneration, and acclimatization) for each parental line increase the cost. However, CRISPR drive carrying a single parental line is sufficient instead of CRISPR on each parental to develop different hybrid cultivars. Homozygous *S*-mutation is brought in different hybrid cultivars by routine pollination without the need for CRISPR reapplication on every hybrid combination. Especially in developing countries, this technology could become accessible for seed companies that cannot afford repetitive CRISPR costs. The seed companies could also corporate CRISPR with breeding programs more productively and effectively, enabling producers to reach seed at a more affordable price.

## Data Availability Statement

The original contributions presented in the study are included in the article/supplementary material, further inquiries can be directed to the corresponding author.

## Author Contributions

MT and KB contributed to putting the idea of using CRISPR drive to generate homozygous *S*-mutant plants and writing this perspective. Both authors contributed to the article and approved the submitted version.

## Conflict of Interest

The authors declare that the research was conducted in the absence of any commercial or financial relationships that could be construed as a potential conflict of interest.

## Publisher’s Note

All claims expressed in this article are solely those of the authors and do not necessarily represent those of their affiliated organizations, or those of the publisher, the editors and the reviewers. Any product that may be evaluated in this article, or claim that may be made by its manufacturer, is not guaranteed or endorsed by the publisher.
